# Digital Droplet PCR for the Absolute Quantification of Exon Skipping Induced by Antisense Oligonucleotides in (Pre-)Clinical Development for Duchenne Muscular Dystrophy

**DOI:** 10.1371/journal.pone.0162467

**Published:** 2016-09-09

**Authors:** Ruurd C. Verheul, Judith C. T. van Deutekom, Nicole A. Datson

**Affiliations:** BioMarin Nederland BV, Leiden, The Netherlands; University of Minnesota Medical Center, UNITED STATES

## Abstract

Antisense oligonucleotides (AONs) in clinical development for Duchenne muscular dystrophy (DMD) aim to induce skipping of a specific exon of the dystrophin transcript during pre-mRNA splicing. This results in restoration of the open reading frame and consequently synthesis of a dystrophin protein with a shorter yet functional central rod domain. To monitor the molecular therapeutic effect of exon skip-inducing AONs in clinical studies, accurate quantification of pre- and post-treatment exon skip levels is required. With the recent introduction of 3^rd^ generation digital droplet PCR (ddPCR), a state-of-the-art technology became available which allows absolute quantification of transcript copy numbers with and without specific exon skip with high precision, sensitivity and reproducibility. Using Taqman assays with probes targeting specific exon-exon junctions, we here demonstrate that ddPCR reproducibly quantified cDNA fragments with and without exon 51 of the DMD gene over a 4-log dynamic range. In a comparison of conventional nested PCR, qPCR and ddPCR using cDNA constructs with and without exon 51 mixed in different molar ratios using, ddPCR quantification came closest to the expected outcome over the full range of ratios (0–100%), while qPCR and in particular nested PCR overestimated the relative percentage of the construct lacking exon 51. Highest accuracy was similarly obtained with ddPCR in DMD patient-derived muscle cells treated with an AON inducing exon 51 skipping. We therefore recommend implementation of ddPCR for quantification of exon skip efficiencies of AONs in (pre)clinical development for DMD.

## Introduction

Duchenne Muscular Dystrophy (DMD) is a severe and progressive muscle-wasting disorder caused by mutations in the *DMD* gene located on chromosome Xp21, which are mostly intragenic deletions (72%) resulting in loss of one or more exons and disruption of the dystrophin open reading frame [1]. There is no dystrophin restoring therapy for DMD available today, although exon skipping therapy using antisense oligonucleotides (AONs), which especially applies to reading-frame disrupting deletions, has advanced to late stage clinical development. Exon skipping AONs are designed to sequence-specifically bind to exon-internal, splicing regulatory sequences and to induce skipping of the targeted exon flanking a deletion during pre-mRNA splicing. The resulting transcript has a restored open reading frame and allows synthesis of a dystrophin protein with a shorter yet functional central rod domain, in many ways similar to the dystrophin protein variants in the typically milder Becker Muscular Dystrophy patients [2–6]. Proof-of-principle of exon skipping has been obtained in healthy donor and DMD patient-derived muscle cell cultures [7–9] various DMD mouse and dog models [10–19] and recently in DMD patients treated with drisapersen or eteplirsen [20–22].

Accurate quantification of induced exon skipping levels is important in the various stages of (pre)clinical development of AONs for DMD; both in the selection of efficient AON candidates in the preclinical phase, and as a pharmacodynamic treatment outcome measure in muscle biopsies from DMD patients participating in clinical trials. Since many DMD patients display a low level of spontaneous, rescuing background exon skipping, accurate quantification of transcripts with and without exon skip before and after AON treatment is important to assess the molecular AON drug effect in clinical studies.

Measurement of exon skipping levels in animal models, DMD patient-derived muscle cell cultures and muscle biopsies has so far mostly been performed using non-quantitative first-generation (nested) PCR in which the same PCR primers are used to co-amplify the original transcript fragment without exon skip and the novel, shorter transcript fragment with exon skip. Since the amplicon resulting from the exon skip is shorter, it is preferentially amplified in the PCR reaction. Depending on the pre-amplification stoichiometry of dystrophin transcripts with and without exon skip, their length difference and the total number of PCR cycles performed in a single or nested PCR reaction, the degree of amplification bias of the shorter product may vary but typically tends to overestimate the levels of induced exon skipping [23].

More recently, 2^nd^ generation real-time quantitative PCR analysis (qPCR) using either SYBR green [24] or specific Taqman assays [25] to quantify the dystrophin transcript fragments with and without exon skip has been explored. The obtained quantification cycle (Cq) values of in particular the shorter transcript fragments with exon skip were however high (>35) and in the range where qPCR becomes less efficient and therefore less reliable. An important aspect of qPCR analysis is the required correction for differences in PCR efficiency between the used Taqman assays or PCR primers. There are multiple ways to determine PCR efficiencies, such as use of a standard curve (also known as the Pfaffl method [26]) or amplification curve analysis (also known as the LinRegPCR method [27]). However, this correction for PCR efficiency may introduce bias and considerably impact the quantification outcome.

**Fig 1:**
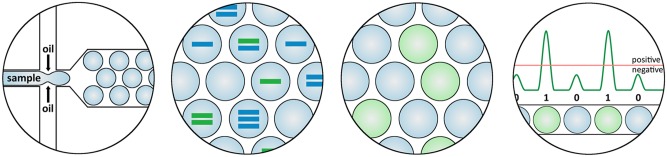
Principle of ddPCR. In ddPCR, a mix of sample and reagents is partitioned into a water-in-oil emulsion consisting of ~20,000 droplets with dilutions of template such that at least some droplets contain no copies of the target of interest. Following end-point PCR amplification, the droplets are screened for fluorescent signal. The outcome is binary, a droplet is either positive (containing target) or negative (no target), based on its fluorescent signal. Quantification of the target concentration is achieved by Poisson correction on the fraction of positive droplets to compensate for the fact that multiple copies of the target may be present in some droplets.

With the recent introduction of 3^rd^ generation digital droplet PCR (ddPCR), a state-of-the-art technology became available which allows absolute quantification of copy numbers of transcripts with and without exon skip with high precision, sensitivity and reproducibility. In ddPCR a standard curve is not required and outcome is not influenced by amplification efficiency. Samples containing template and PCR reagents are partitioned into a water-in-oil emulsion consisting of ~20,000 droplets and are subjected to PCR cycling, with each droplet representing an individual micro PCR reaction. Template molecules are randomly divided over the individual droplets and diluted so that at least some droplets contain no copies of the target of interest. After cycling, droplets are screened for fluorescent signal with a binary outcome, a droplet is either positive or negative for fluorescent signal (Fig 1). Therefore the amplification efficiency of the amplicon is not relevant, in contrast to qPCR where the fluorescent signal should increase exponentially per cycle. Due to the partitioning, many thousands of data points are obtained per ddPCR reaction, providing a much more detailed and powerful measurement than in qPCR and allowing precise quantification of the number of template molecules using Poisson statistics. The more droplets that are screened per sample, the more accurate the quantification of template copy number is, which can easily be achieved by running multiple parallel replicates. ddPCR allows precise determination of absolute copy numbers of transcripts with and without exon skip and therefore accurate calculation of exon skip percentages.

We have developed Taqman assays with probes targeting specific exon-exon junctions in *DMD* transcripts with and without skipping of exon 51 and have used ddPCR to quantify levels of exon 51 skip. Here we demonstrate that quantification of exon skip levels using ddPCR is most accurate in comparison to conventional PCR and qPCR and does not overestimate exon skip levels. In addition, we show that ddPCR is highly sensitive and reproducible and able to quantify even very low levels of spontaneous background exon skip. Finally, we demonstrate the applicability of ddPCR in the accurate quantification of exon skip levels in myogenic and muscle cell cultures with different mutational backgrounds amenable to exon 51 skipping.

## Materials and Methods

### Myogenic and muscle cell lines

Immortalized DMD patient-derived myoblasts with a deletion of exons 48–50 (Δ48–50) were kindly provided by the Association Institut de Myology (AIM) [28]. Skin biopsies of DMD patients participating in a clinical study (registered at clinicaltrials.gov; NCT01910649) were used to derive primary fibroblasts with a deletion of exons 45–50 (Δ45–50), exons 49–50 (Δ49–50), exon 50 (Δ50) or exon 52 (Δ52) as previously described [29]. Permission was obtained from the BioMarin Nederland BV internal medical ethics oversight committee and signed patient informed consent was given, after which cell lines were blinded and used for research purposes. Experiments were performed in accordance with European recommendations and Dutch legislation.

### Cell culture and AON transfection

Immortalized DMD patient-derived myoblasts (Δ48–50) were cultured in collagen coated flasks or 6-well plates. Upon confluency myoblast cultures were serum deprived for 3–5 days in order to induce myotube formation.

DMD patient-derived fibroblasts (Δ45–50, Δ49–50, Δ50 and Δ52) were grown to 75–90% confluency before transduction using adenoviral vector Ad5fib50-MyoD [30]. The cells were serum deprived for 5–10 days in order to induce myotube formation. Myotube cultures were subsequently transfected with 400nM of ex51 AON, an exon 51 2′-*O*-methyl phosphorothioate AON [8] using polyethylenimine (PEI). Typically, cells were incubated with 2 μl PEI/μg AON for 4 hours after which fresh culture medium was added. Total RNA was isolated 24 hours post-transfection. No PEI was added to the non-treated cells.

### Dystrophin cDNA constructs

Dystrophin cDNA constructs, representative for the transcript arising from an exon 48 to 50 deletion (containing a partial fragment of exon 44 and exons 45-46-47-51-52-53-54) and the resulting transcript fragment following exon 51 skipping (with a partial fragment of exon 44, exons 45-46-47-52-53-54 and a partial fragment of exon 55) were synthesized by Life Technologies. Both cDNA constructs had a length of 1200 bp and were verified by sequencing. Starting from equimolar stock solutions, different dilutions and ratios of both cDNA constructs were used for temperature gradient, reproducibility and dilution linearity experiments. Furthermore, different ratios of the cDNA constructs were used as input in nested PCR, qPCR and ddPCR to compare the different exon skip quantification methodologies. Similar dystrophin cDNA constructs representative for other exon 5skip amenable deletions were also designed, all with a 1200 bp insert length (S1 Table).

### RNA isolation and cDNA synthesis

Total RNA was extracted from AON treated or untreated induced myogenic or muscle cell cultures using RNA-Bee (#CS-105B, Bio-Connect) according to the manufacturer’s instructions. cDNA was generated in 20 μl reactions, using 1000 ng of total RNA with random hexamer primers (#11034731001, Roche) and Transcriptor Reverse transcriptase (#3531287001, Roche) according to the manufacturer’s instructions.

### Primary and nested PCR analysis

A primary PCR was performed for 20/30/40 cycles on 3 μl of cDNA in a 25 μl reaction volume with Taq polymerase (#11596594001, Roche) and using primers flanking each deletion (S2 Table). The following protocol was used: 94°C for 5 min, 20/30/40 cycles of 94°C for 40 sec, 60°C for 40 sec and 72°C for 80 sec, followed by 72°C for 7 min. Next, 1.5 μl of the primary PCR mixture was re-amplified for 20/24/30/40 cycles in a 50 μl nested PCR using primers internal to those used in the primary PCR (94°C for 5 min, 20/24/30/40 cycles of 94°C for 40 sec, 60°C for 40 sec and 72°C for 60 sec, followed by 72°C for 7 min). In both primary and nested PCR run, a no template control (NTC) was included as negative control. PCR products were visualized on 2% agarose gels and semi-quantitative analysis was performed using the Lab-on-a-Chip analysis system and the 2100 Bioanalyser (Agilent). The DNA concentration (in nM) of the skipped band and non-skipped band was used to calculate the percentage exon skip [nM skipped/(nM skipped + nM non-skipped)*100].

### Taqman Assays

**Fig 2:**
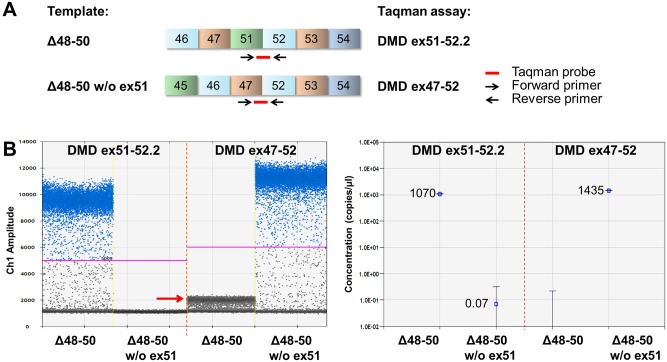
Design and specificity of the Taqman assays representative for the exon 48–50 deletion transcripts in ddPCR analysis. (A) Position of Taqman MGB assays that were designed to detect exon 51 skipping in a muscle cell culture from a DMD patient with a deletion of exons 48–50 (Δ48–50). Primers and exon spanning probes were positioned as indicated by the arrows and the red lines. (B) The specificity of both DMD ex51-52.2 and DMD ex47-52 Taqman assays was confirmed by ddPCR analysis using dystrophin cDNA constructs representative for Δ48–50 transcript fragments with or without exon 51. The left panel shows the droplet populations obtained using both assays. The ex51-52.2 assay generated positive droplets when the construct containing exon 51 was used as template, but not when the exon 51 lacking construct was the template. For the ex47-52 assay detection was vice versa, yielding positive droplets using the exon 51 lacking template. The red arrow (in left panel) indicates a cloud of droplets with low fluorescence, arising from partial binding of the Taqman probe used in this assay to exon 47 and/or 52 sequences present but not joined in the template containing exon 51. The right panel shows the concentration of the constructs that was calculated based on the number of positive droplets for both assays. Data is represented as mean with 95% confidence interval (CI) (right panel).

Specific Taqman minor groove binder (MGB) assays were designed to detect the dystrophin products with and without exon 51 (Fig 2A). Taqman MGB assays were designed using Primer Express 3.0.1 software (Applied Biosystems) and purchased from Applied Biosystems (S2 Table). All Taqman assays were used with an annealing/extension temperature of 60°C.

### Real time quantitative PCR analysis

Real time quantitative PCR (qPCR) was performed in triplicate on 2 μl of cDNA in a 10 μl reaction volume containing 0.5 μl of the relevant Taqman assay, 5 μl of Gene expression master mix (#4369016, Applied Biosystems) and 3 μl of DNase/RNase-free H_2_O. cDNA fragments with and without exon 51 skip were detected in separate reactions. In each qPCR run, a no template control (NTC) was included as negative control. cDNA fragment levels were determined on a CFX96 real-time thermal cycler (BioRad) using the following protocol: 95°C for 10 min, then 40 cycles of 95°C for 15 sec and 60°C for 1 min. qPCR data was analyzed using both the Pfaffl method [26] and the LinRegPCR method [27]. The Pfaffl calculation was performed using the efficiencies derived from the standard curves for both assays (with and without exon 51 skip) in the following manner: ratio of skipped versus non-skipped = E (skipped)^-Cq (skipped)^/E (non-skipped)^-Cq (non-skipped)^. Data was expressed as percentage exon skip [ratio skipped/(ratio skipped+1)]. The LinRegPCR method, which uses linear regression analysis to determine the PCR efficiency per sample based on the slope of the exponential phase of the amplification curve, required the raw fluorescent data (not baseline corrected) to be imported in LinRegPCR software version 2014.6. The mean PCR efficiency per amplicon (E) was used to calculate a starting concentration per sample (N_0_), expressed in arbitrary fluorescence units using the equation N_0_ = N_t_/E^Cq^ where N_t_ is the fluorescence threshold and Cq is the cycle number per sample [27]. Data was presented as percentage exon skip [N_0_ skipped/(N_0_ skipped + N_0_ non-skipped)*100].

### Digital droplet PCR (ddPCR)

Digital droplet PCR (ddPCR) was performed on 2 μl of cDNA in a 20 μl reaction volume containing 1 μl of the relevant Taqman assay, 10 μl of ddPCR Super mix for probes (#186–3010, BioRad) and 7 μl of DNase/RNase-free H_2_O. In each ddPCR run multiple technical replicates were run in parallel and a no template control (NTC) was included as negative control. Each 20 μl PCR reaction was loaded in an 8-channel disposable droplet generation cartridge. Seventy μl of droplet generation oil (#186–3005, BioRad) was loaded in adjacent oil wells on the cartridge and the microfluidic chip was placed in the QX200 droplet generator (BioRad). During this process the sample mix was partitioned into ~20,000 nano droplets of equal size, randomly distributing target molecules into droplets. The resulting water-in-oil emulsion was pipetted from the outlet well to a semi-skirted 96 well PCR plate (#0030 128.591, Eppendorf). After transfer of all samples, the plate was sealed with foil and amplified on a T100 thermal cycler (BioRad) using the following protocol: 95°C for 10 min, 40 cycles of 95°C for 15 sec and 60°C for 1 min, and 98°C for 10 min to inactivate enzymatic nuclease activity. Plates containing the amplified droplets were loaded into the QX200 droplet reader (BioRad), which aspirates droplets from the 96-well plate, one well at a time, and streams them in single file past a detector for FAM and VIC/HEX fluorescent dyes. On average data from 12,000–18,000 droplets per sample was used in subsequent calculations.

Discrimination between droplets without target (negatives) and droplets with target (positives) was achieved by applying a fluorescence amplitude threshold. For both the DMD ex47-52 and DMD ex51-52 Taqman assays, a threshold of 4,000–5,000 relative fluorescent units (RFU) was applied, but was reset manually to a different value if this gave a better discrimination between the positive and negative droplet population. Digital PCR runs were considered technically successful if the number of accepted droplets per individual sample replicate was >10,000. To assess variability between technical replicates the coefficient of variation (%CV = (stdev/avg)*100) was calculated. The concentration (in copies/μl sample mix) of the skipped assay and non-skipped assay was used to calculate the percentage exon skip [copies/μl skipped/ (copies/μl skipped + copies/μl non-skipped)*100].

## Results

### Quantification by ddPCR is not influenced by PCR amplification efficiency

**Fig 3:**
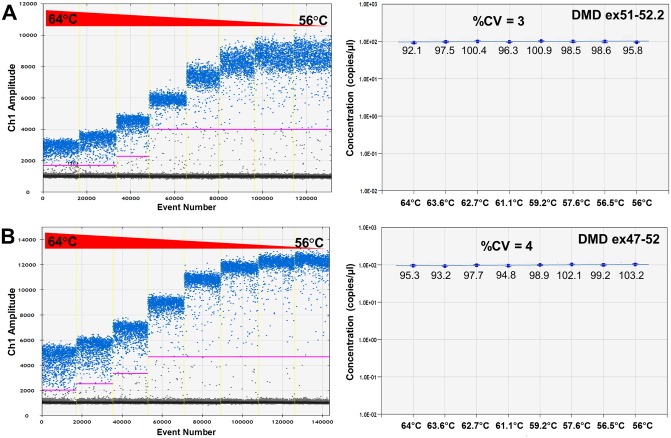
Temperature gradient ddPCR for both DMD ex51-52.2 and DMD ex47-52 assays. (A) DMD ex51-52.2 assay and (B) DMD ex47-52 assay. The left panels show how the positive droplet populations change with decreasing annealing temperatures. At higher annealing temperatures the overall fluorescence of the positive droplet population (in blue) is lower, caused by a less efficient PCR amplification, and consequently the separation of the positive and negative droplet (in grey) clouds is less pronounced. In the right panels the corresponding concentrations of the constructs along the temperature gradient are represented. The %CV (coefficient of variation) between the different annealing temperatures is 3–4%, indicating a highly consistent quantification of the construct concentration, even at suboptimal amplification conditions at then higher annealing temperatures. Data is represented as mean with 95% CI (right panel).

Taqman assays were designed to specifically detect *DMD* cDNA fragments with (DMD ex51-52.2) or without exon 51 (DMD ex47-52) in a DMD mutational background of an exon 48–50 deletion (Δ48–50), which occurs in ~7% of the DMD deletions [31]. The specificity of these assays was confirmed in immortalized patient cells (Δ48–50) by qPCR and subsequent agarose gel analysis (S1A and S1B Fig) and by ddPCR analysis using dystrophin cDNA constructs with and without exon 51 as template (Fig 2A). As expected, the DMD ex51-52.2 assay only detected the template with exon 51 and the DMD ex47-52 only detected the template without exon 51 (Fig 2B). An additional cloud with low fluorescent positive droplets was obtained just above background signal when the template containing exon 51 was measured with the DMD ex47-52 assay (Fig 2B). This droplet population (indicated by arrow in Fig 2B) arises from partial binding of the Taqman probe used in this assay to exon 47 and/or 52 sequences that are also present but not joined in the template containing exon 51, resulting in increased background fluorescence. However, as the fluorescent threshold was automatically set well above this background droplet population, the measurement was not influenced by this non-specific binding. To mimic different amplification efficiencies, annealing/extension temperature gradient ddPCRs were performed for both Taqman assays. For both the DMD ex51-52.2 and DMD ex47-52 assay, a better separation of positive and negative droplets and a higher fluorescent signal arising from a more efficient PCR amplification was observed with lower annealing/extension temperatures (Fig 3). Nevertheless, highly similar product concentrations were measured across the entire temperature range, indicated by the low coefficient of variation (%CV) of 3% for the DMD ex51-52.2 assay (Fig 3A) and of 4% for the DMD ex47-52 assay (Fig 3B). This demonstrates that even at suboptimal annealing temperatures leading to lower fluorescence intensities of the positive droplet fraction, the quantification outcome by ddPCR is highly consistent and thus not influenced by PCR amplification efficiency.

### ddPCR analysis is reproducible over a 4-log dynamic range of template concentrations

**Fig 4:**
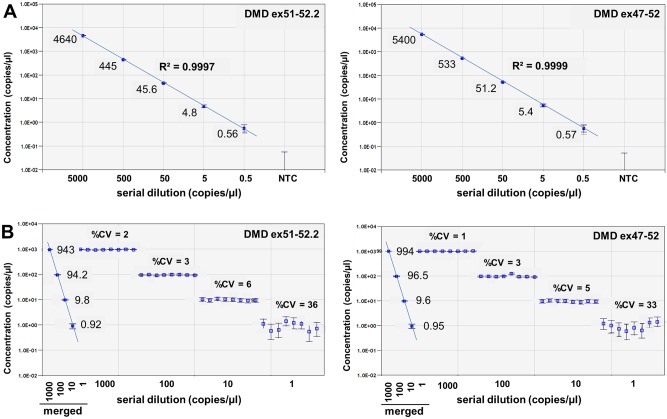
Dilution linearity and reproducibility of ddPCR results. (A) ddPCR analysis was performed on a serial dilution of dystrophin cDNA templates representative for the Δ48–50, with or without exon 51, using Taqman assays with a probe detecting the exon 51–52 junction (DMD ex51-52.2) or exon 47–52 junction (DMD ex47-52) respectively. Concentrations ranged from >5,000 copies/μl down to <1 copy/μl. Dilution linearity is indicated by the coefficient of determination (R^2^). (B) ddPCR analysis of 8 technical replicates at different template copy numbers (from 1 to 1,000 copies/μl) using the DMD ex51-52.2 and DMD ex47-52 Taqman assays. Merged data of 8 replicates is indicated by solid markers. Data is represented as mean with 95% CI.

To demonstrate the reproducibility of ddPCR quantification over a wide dynamic range of template concentrations, 10-fold serial dilutions of dystrophin cDNA constructs with and without exon 51 were generated and used as template in ddPCR analysis using the DMD ex47-52 and DMD ex51-52.2 assays (Fig 4A). Using 4 replicates per concentration, both assays were linear over a 4-log dynamic range from 5,000 copies/μl to concentrations as low as 0.5 copies/μl.

To determine the precision of the DMD ex51-52.2 and DMD ex47-52 ddPCR assays, different concentrations of the dystrophin cDNA constructs with and without exon 51 were measured in 8 replicates per concentration (Fig 4B). The observed variation between replicates was very low, especially at template concentrations of at least 10 copies/μl (%CV<6). At lower template concentrations of approximately 1 copy/μl, the variation between replicates was higher (%CV 33–36), which is largely due to the increased effect of subsampling error in the poisson-based calculation model. However, when data from all 8 replicates was merged into a single data point, the reliability of the measurements improved and the 95% confidence interval (95% CI) decreased, as indicated by smaller error bars (Fig 4B). Therefore, by increasing the number of replicates and thus the number of analyzed droplets (sample size) for measurements at low template concentrations, the reported 95% CI and reliability of the measurement can be improved, allowing better discrimination in case of small differences in levels of template molecules.

### Comparative quantification of ratios of exon 51 lacking/containing constructs using different PCR methods

**Fig 5:**
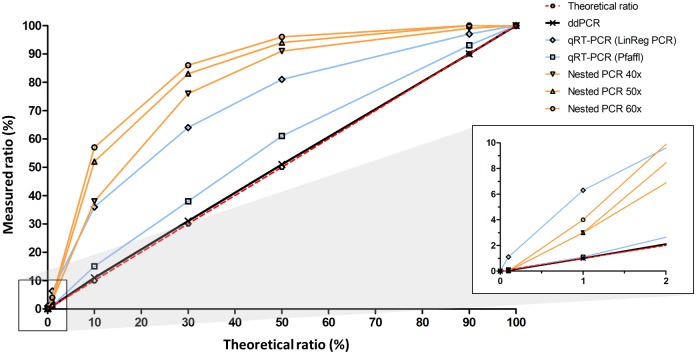
Comparison different PCR methods and impact on quantification. Graphic representation of the quantification of dystrophin cDNA construct templates lacking or containing exon 51 mixed in different ratios ranging from 0% to 100% using nested PCR (40, 50 and 60 cycles), qPCR (LinRegPCR and Pfaffl methods) and ddPCR. The dashed red line indicates the theoretical template ratios. The solid black ddPCR line is almost indistinguishable from the theoretical line and both lines are superimposed. Data is based on one experiment using 2 replicates for ddPCR and 3 replicates for qPCR.

The accuracy of exon 51 skip quantification using ddPCR was compared with qPCR and conventional nested PCR. To mimic different percentages of exon 51 skip in an amenable deletion background, partial dystrophin cDNA constructs with an exon content representative of an exon 48–50 deletion with and without exon 51 skipped were constructed, mixed at molar ratios ranging from 0% to 100% and used as a template in the different PCR reactions. ddPCR analysis of the *DMD* cDNA construct template mixtures using the DMD ex47-52 and the DMD ex51-52.2 assays yielded an outcome highly identical to the expected percentages based on molar ratio of the mixed constructs. This was the case over the entire range of template mixtures, demonstrating the quantification accuracy of ddPCR (Table 1 and Fig 5).

**Table 1:** Quantification of ratios of exon 51 lacking/containing constructs using different PCR methods.

Theoretical ratio	ddPCR	qPCR (Pfaffl)	qPCR (LinRegPCR)	Nested PCR 40 cycles	Nested PCR 50 cycles	Nested PCR 60 cycles
0.0%	0.0%	0.0%	0.0%	N/A	N/A	N/A
0.1%	0.1%	0.1%	**1.1%**	**0.0%**	**0.0%**	**0.0%**
1%	1%	1%	**6%**	**3%**	**3%**	**4%**
10%	11%	**15%**	**36%**	**38%**	**52%**	**57%**
30%	31%	**38%**	**64%**	**76%**	**83%**	**86%**
50%	51%	**61%**	**81%**	**91%**	**94%**	**96%**
90%	90%	93%	97%	99%	**100%**	**100%**
100%	100%	100%	100%	100%	100%	100%

Ratios that deviate more than 10% from the theoretical values are underscored and highlighted in bold. N/A indicates that no valid measurement was obtained. Data is based on one experiment using 2 replicates for ddPCR and 3 replicates for qPCR.

For qPCR analysis, the same DMD ex51-52.2 and DMD ex47-52 assays were used as for ddPCR. First, their specificity in a qPCR set-up was confirmed using the dystrophin cDNA constructs described above. To determine the qPCR efficiencies of both assays, standard curves were prepared using a 10-fold serial dilution of a 50–50 mixture of the dystrophin cDNA construct templates. Both assays showed good linearity and had efficiencies of at least 94% (S2 Fig). Subsequently, cDNA construct mixtures were used as template in qPCR reactions and ratios of the products lacking or containing exon 51 were calculated. Data was analyzed using both the Pfaffl method [26] and the LinRegPCR method [27] (Table 1 and Fig 5). The Pfaffl calculation was based on the amplification efficiencies derived from the standard curves for both DMD ex47-52 (94.2%) and DMD ex51-52.2 (97.8%) assays. Ratios calculated with this method showed a moderate (1.5 fold) overestimation of the ratio of exon 51 lacking/containing constructs. When quantification was performed using the LinRegPCR method, which uses linear regression analysis to determine the PCR efficiency per sample based on the slope of the exponential phase of the amplification curve, the resulting construct ratios were higher over the entire range and even 6 to 10-fold higher than expected at the low end where ratios were ≤ 1%.

Finally, the same template mixtures were analyzed with conventional non-quantitative nested PCR using a total number of 40, 50 or 60 amplification cycles, and the Lab-on-a-Chip technology. Ratios of exon 51 lacking/containing constructs were 1.8- to 5.7-fold higher than expected based on the molar ratio of the mixed constructs (Table 1 and Fig 5). This overestimation was more pronounced as the total number of PCR cycles increased and highest at ratios between 1% and 30%. Nested PCR was not able to detect skip levels of 0.1% or lower.

In summary, quantification of exon 51 lacking/containing construct ratios by ddPCR outperformed the other PCR methodologies over the entire range of molar ratios, with hardly any deviation from expected values. Conversely, the quantification by nested PCR using a total of 60 PCR cycles resulted in the highest overestimation, in particular at ratios below 30%.

### Comparative quantification of exon 51 skip levels in DMD patient-derived muscle cells using different PCR methods

**Fig 6:**
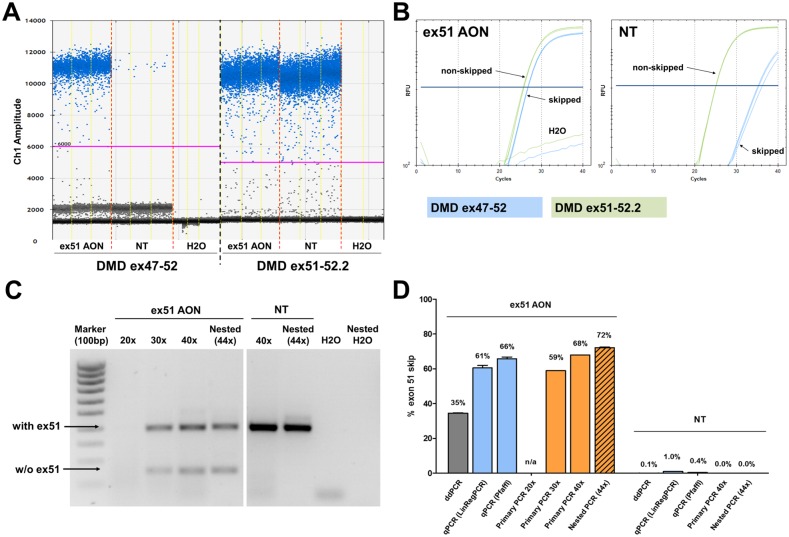
Comparison of different PCR methods on patient-derived muscle cell cultures. Quantification of exon 51 skip levels in patient-derived muscle cell cultures (Δ48–50), transfected with an exon 51 skipping AON (400nM) by (A) ddPCR analysis, (B) qPCR analysis and (C) conventional primary PCR (20/30/40 cycles) and nested PCR (44 cycles). (D) Low background levels of exon 51 skipping were detected by ddPCR and qPCR in the non-treated (NT) samples. N/a indicates that no exon skip percentage could be calculated. Percentages shown are mean (n = 3) with SEM.

Exon 51 skip levels were quantified in immortalized patient-derived muscle cell cultures from a patient with an exon 48 to 50 deletion (Δ48–50) transfected with ex51 AON (Fig 6). Total RNA was isolated at 24 hours post-transfection and random hexamer primers were used to generate cDNA in triplicate. These technical replicates were used as input for the different analyses. ddPCR analysis was performed with the DMD ex51-52.2 and DMD ex47-52 Taqman assays using three replicates per amplicon (Fig 6A). Exon 51 skip levels were approximately 35% for AON treated cells and 0.1% for non-treated (NT) cells (Fig 6D). qPCR analysis using the same DMD ex51-52.2 and DMD ex47-52 Taqman assays and the same cDNA as input yielded exon 51 skip levels that were respectively 1.8-fold higher using the Pfaffl method and 1.9-fold higher using the LinRegPCR method than those obtained with ddPCR (Fig 6B–6D). Conventional primary PCR analysis (20/30/40 cycles) and nested PCR analysis applying a total of 44 PCR cycles yielded exon skip levels which were up to 2-fold higher than those obtained with ddPCR (Fig 6C and 6D). Furthermore, the spontaneous background exon 51 skipping events in non-treated cells were clearly detected by ddPCR, but not by primary PCR or nested PCR, underlining the high sensitivity of the ddPCR analysis.

### ddPCR Taqman assays for other deletions amenable to exon 51 skipping

**Fig 7:**
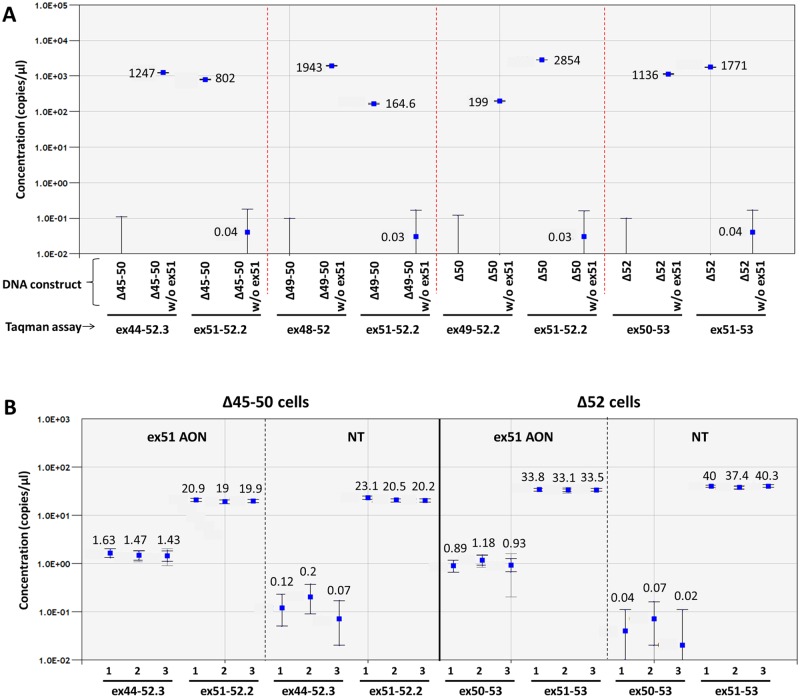
Taqman assay for 4 additional deletions. The specificity of Taqman assays with a probe detecting the exon junctions with (DMD ex44-52.3/DMD ex48-52/DMD ex49-52.2/DMD ex51-53) and without (DMD ex51-52.2/DMD ex51-53) exon 51 skip for four additional relatively frequent deletions amenable to exon 51 skipping (Δ45–50, Δ49–50, Δ50, and Δ52) was confirmed on representative dystrophin cDNA constructs with or without exon 51 (A). Exon 51 skip quantification in MyoD converted patient fibroblasts (Δ45–50 and Δ52) with and without ex51 AON treatment in triplicate (B). Merged data of 3 replicates is indicated by solid markers. Data is represented as mean with 95% CI (dark grey error bars: poisson CI; light grey error bars: total CI).

Additional Taqman assays were developed for four other deletions amenable to exon 51 skipping: Δ45–50, Δ49–50, Δ50, and Δ52 (S3 Fig). The specificity of these assays was confirmed by gel electrophoresis in MyoD transduced patient derived fibroblasts with different mutational backgrounds (Δ45–50, Δ49–50, Δ50 and Δ52) transfected with ex51 AON (S1C Fig) and by ddPCR on representative dystrophin cDNA constructs using three replicates per amplicon (Fig 7A). As expected, the assays were specific for either the construct with or without exon 51, depending on their design. In both the Δ45–50 and Δ52 cell lines, transcripts with and without exon 51 skipping could be reproducibly detected by ddPCR (Fig 7B). Transcripts lacking exon 51 were detected in samples treated with the AON, but also at much lower levels (150–1000 fold) in the non-treated samples. This is most likely the result of spontaneous background exon 51 skip, which seems to occur at a slightly higher rate in the Δ45–50 than in the Δ52 cells. Overall, in comparison with the immortalized Δ48–50 cells, much lower concentrations of both the transcript with and without exon 51 were found. Moreover, the exon skip levels (7% in the Δ45–50 and 3% in the Δ52 cell line respectively) were also lower than in the immortalized Δ48–50 cells, probably due to the relatively inefficient MyoD conversion of myoblasts to dystrophin expressing myotubes compared to the immortalized DMD patient cell line. However, differences in mutational background may also influence exon skip efficiency.

## Discussion

Targeted exon skipping using antisense oligonucleotides (AONs) to correct reading frame disrupting deletions is a therapeutic approach that is in late stage clinical development for DMD. Since this is a mutation-dependent approach, several different AONs are required to target the majority of the DMD mutations occurring in the mutational hotspot region between exons 45 and 55. Drisapersen and eteplirsen are designed for DMD patients whose mutation is amenable to exon 51 skipping, constituting approximately 13% of the DMD population. While the primary endpoint to assess efficacy in clinical trials is the six minute walk test [32], dystrophin protein expression is also monitored as an outcome measure in patient muscle biopsies using western blotting or immunocytochemistry [33, 34]. However, dystrophin quantification has not yet risen to the standard required of a surrogate marker, namely that changes in expression are predictive of changes in clinical outcome variables. In a recent study in BMD patients with varying levels of dystrophin expression (3%-78% of healthy control), no relation was found between levels of dystrophin and muscle strength or age [35]. This illustrates that the biology of dystrophin and how much is required to improve or preserve muscle function still remains not fully understood [36–38].

Since the primary anticipated mechanism of action of AONs for DMD is induction of exon skipping, measuring exon skip levels in muscle biopsies from treated patients may serve as a supportive molecular marker. However, as a majority of DMD patients have a low level of spontaneous skipping of exons flanking their deletions, accurate quantification of exon skipping levels in pre- versus post-treatment biopsies is essential. Unfortunately, the conventional nested PCR method so far widely used in the exon skipping field to measure exon skip levels is qualitative or at best semi-quantitative and has resulted in a systematic and widespread overestimation of exon skip levels.

Recently, due to technological advances improved PCR-based methodologies have become available. Here we show that third generation ddPCR is an extremely sensitive, reproducible and reliable method for absolute quantification of exon skip levels in (pre)clinical samples. We have demonstrated that ddPCR can reliably quantify exon skip levels over a wide 4-log dynamic range of template concentrations and that the outcome is very robust and not influenced by differences in PCR efficiency. It is worth noting that ddPCR is not more time consuming than conventional nested PCR followed by lab-on-chip analysis. Furthermore, ddPCR is highly sensitive and can detect a very low level of spontaneous exon 51 skipping occurring at baseline in a DMD muscle cell culture. To allow applicability of this methodology for muscles biopsy analysis in clinical studies, we have developed Taqman assays for 5 different DMD deletions amenable to exon 51 skipping. Together these mutations represent the majority of the total number of DMD deletions correctable by exon 51 skipping. We have demonstrated the specificity of all these assays on cDNA constructs that reflect the exon content of the different mutations with and without exon 51 skipping. In addition, we have also quantified exon 51 skip levels in vitro in patient derived muscle cell cultures transfected with ex51 AON. When comparing the ability of different PCR-based methods to accurately quantify different molar ratios of cDNA constructs with and without exon 51, ddPCR came closest to the theoretical calculated outcome over the full range of ratios (0–100%). In contrast, qPCR overestimated the exon skip levels in particular when the LinRegPCR method was applied to correct for differences in PCR efficiency between the assays for the cDNA constructs with and without exon 51. This can largely be explained by the difference in mean efficiencies between the assays calculated by the LinRegPCR software. The mean efficiency of the DMD ex47-52 assay was calculated to be approximately 11% lower than that of the DMD ex51-52.2 assay, resulting in an overestimation of the exon 51 lacking/containing construct ratio. This overestimation of exon skip was even more pronounced using nested PCR, and increased with the total number of PCR cycles, probably due to amplification bias towards the shorter amplicon.

Although ddPCR offers a precise, reproducible and absolute quantification of exon skip levels, the measurement is similarly impacted by several upstream factors that may influence outcome and results should always be interpreted carefully taking technical and biological limitations into account. For example, muscle biopsies obtained from DMD patients often have a lower muscle fiber content and more fibrotic or fatty tissue than in healthy donor biopsies, resulting in lower levels of non-skipped dystrophin expression and potentially hampering reliable quantification of exon skip levels. In addition, it is not always feasible to isolate high quality RNA from patient biopsies, which may lead to increased variation. Furthermore, efficiency of the reverse transcription step to convert RNA to cDNA may introduce variation in ddPCR measurements. Since different cDNA priming methods are known to generate different outcomes [39], use of generic random hexamer primers that can be applied to all mutations and templates facilitates comparison of exon skip levels in different mutational backgrounds. Finally, since AON uptake, exon skip levels and subsequent dystrophin restoration likely vary between different muscle groups and do not occur homogeneously even within a specific muscle [40], a single muscle biopsy can never fully reflect the therapeutic effect of exon skipping on the entire muscle compartment of the body.

Another consideration to take into account when monitoring exon skip levels using *DMD* exon specific primers, probes and assays is that the complexity of the dystrophin transcriptome can never be entirely covered using Taqman assays or PCR amplicons that specifically detect or amplify a single exon-exon junction. The dystrophin pre-mRNA is subject to extensive alternative splicing [41], which generates a series of alternative transcripts that may remain undetected by the used assays. Furthermore, a prominent 5′–3′ non-horizontal representation of *DMD* transcript levels has been reported to occur in skeletal muscles of *mdx* mice and in patients with Becker muscular dystrophy, with reduced transcript levels toward the 3′ end [24]. This 5′-3′ dystrophin transcript imbalance may impact on quantification of exon skip levels in DMD patients. Therefore, quantifying exon skip levels by measuring at a single location in the transcript represents a snapshot of the dystrophin transcriptome and may not necessarily reflect the totality of the molecular treatment effect.

Despite these limitations that more generally apply to working with patient biopsies, the actual quantification of exon skip levels in a given cDNA sample using ddPCR has a high precision, sensitivity and reproducibility and importantly does not overestimate exon skip. Since accurate detection of increased exon skipping levels in post-treatment biopsies would be supportive for an AON drug effect, we thus recommend ddPCR technology for quantification of exon skip efficiencies in the various stages of (pre)clinical development of AONs for DMD.

## Supporting Information

S1 Fig Specificity of Taqman assays in cells. Specificity of the Taqman assays representative for the exon 48–50 deletion transcripts in qPCR analysis and subsequent gel electrophoresis. (A) The specificity of both DMD ex51-52.2 and DMD ex47-52 Taqman assays was confirmed by qPCR analysis using cDNA from a Δ48–50 cell line. The DMD ex47-52 assay detected the skipped product in treated cells, whereas nothing was detected in NT samples. (B) Gel analysis of qPCR products for both DMD ex47-52 and DMD ex51-52.2 Taqman assays. Primers for the DMD ex47-52 Taqman assay also amplify the transcript in which exon 51 is still included, resulting in two bands differing in size by approximately 200 bp, which corresponds to the size of exon 51 (233bp). However, specificity for the skipped product is provided by the Taqman probe directed against the exon 47–52 junction, which only generates a fluorescent signal for the smaller product lacking exon 51(C). Gel analysis of qPCR products for Taqman assays that detect skipped transcripts (DMD ex44-52.3, DMD ex48-52, DMD ex49-52.2 & DMD ex50-53) and non-skipped transcripts (DMD ex51-53) in ex51 AON treated patient derived myotubes with different mutational backgrounds. DMD ex44-52.3, DMD ex48-52, DMD ex49-52.2 & DMD ex50-53 assays all show two products separated by ~233 bp (the size of exon 51): the transcript without exon 51 (lower band) and the transcript containing exon 51 (upper band). Specificity for the skipped product is provided by the Taqman probe which which only generates a fluorescent signal for the smaller product lacking exon 51.
(TIF)

S2 Fig Optimization of Taqman assays for qPCR analysis. Specificity and efficiency of the Taqman assays representative for the exon 48–50 deletion transcripts in qPCR analysis. (A) The specificity of both DMD ex51-52.2 and DMD ex47-52 Taqman assays was confirmed by qPCR analysis using dystrophin cDNA constructs representative for Δ48–50 transcript fragments with or without exon 51. (B) 10-fold serial dilution of a 50–50 mixture of the dystrophin cDNA construct templates representative for Δ48–50 transcript fragments with or without exon 51.
(TIF)

S3 Fig Panel of Taqman assays for most common exon 51 skip amenable deletions. Taqman assays were designed to detect exon 51 skipping in samples of DMD patients with different relevant deletions using the guidelines of the Primer express software (Applied Biosystems). For the specific detection of transcripts containing exon 51, the forward primer was located in exon 51 and the reverse primer in exon 52, while the Taqman probe spans the exon-junction. For the samples with an exon 52 deletion, an alternative assay was designed to detect the transcript containing exon 51.
(TIF)

S1 Table Design of dystrophin cDNA constructs for exon 51 skip amenable deletions. Dystrophin cDNA constructs, representative for the transcripts arising from several exon 51 skip amenable deletions and the resulting transcript fragment following exon 51 skipping, were synthesized by Life Technologies. All cDNA constructs had a length of 1200bp and were verified by sequencing. Dark shaded exons were included entirely and light shaded exons were partially included.
(PDF)

S2 Table Sequences of PCR primers and Taqman probes. (PDF)
