# Supplementary figures and images for: Digital Droplet PCR for the Absolute Quantification of Exon Skipping Induced by Antisense Oligonucleotides in (Pre-)Clinical Development for Duchenne Muscular Dystrophy
Source: PLoS One. 2016 Sep 9;11(9):e0162467. doi: 10.1371/journal.pone.0162467 (PMC5017733; doi:10.1371/journal.pone.0162467)

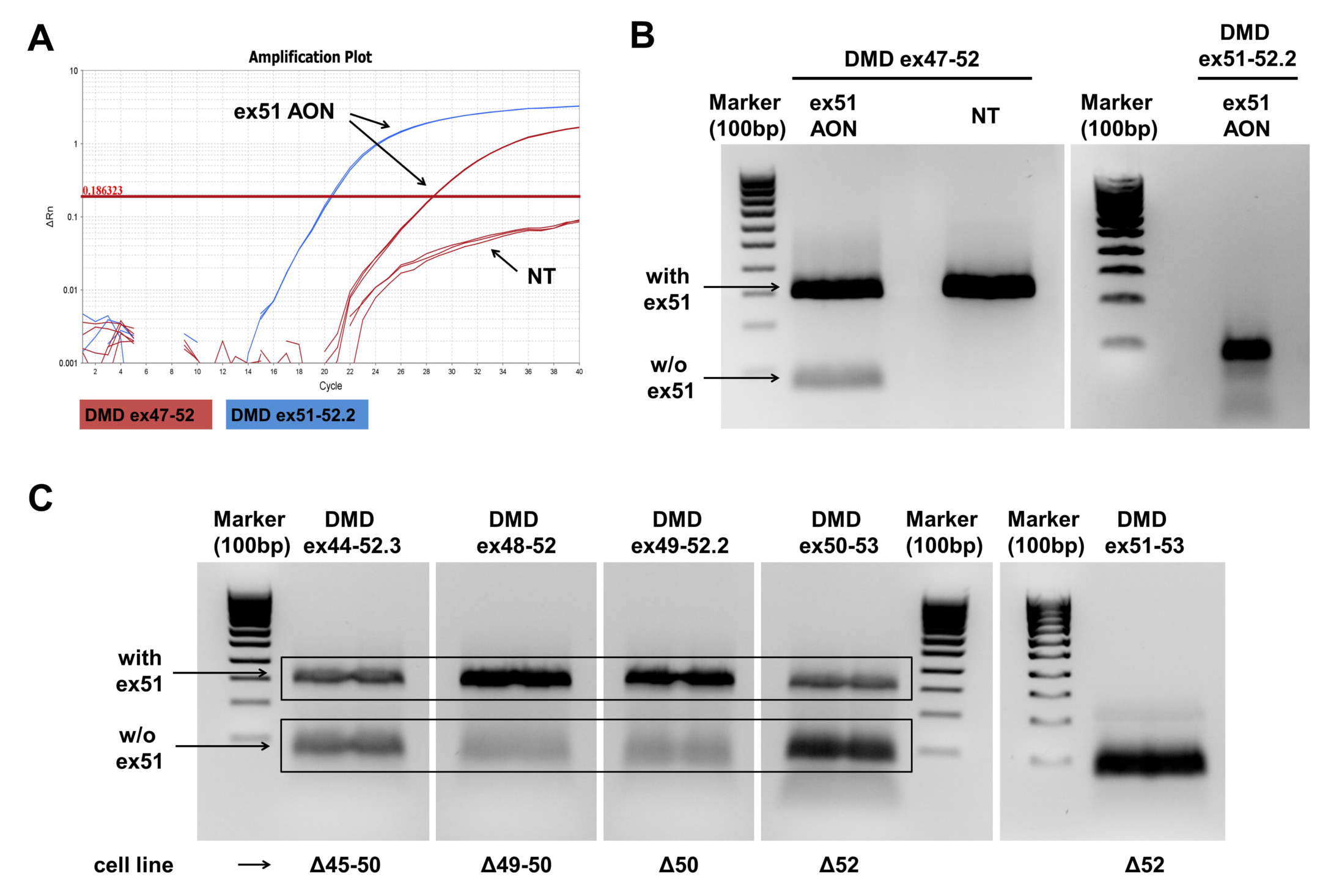

Supplement: S1 Fig — Specificity of the Taqman assays representative for the exon 48–50 deletion transcripts in qPCR analysis and subsequent gel electrophoresis. (A) The specificity of both DMD ex51-52.2 and DMD ex47-52 Taqman assays was confirmed by qPCR analysis using cDNA from a Δ48–50 cell line. The DMD ex47-52 assay detected the skipped product in treated cells, whereas nothing was detected in NT samples. (B) Gel analysis of qPCR products for both DMD ex47-52 and DMD ex51-52.2 Taqman assays. Primers for the DMD ex47-52 Taqman assay also amplify the transcript in which exon 51 is still included, resulting in two bands differing in size by approximately 200 bp, which corresponds to the size of exon 51 (233bp). However, specificity for the skipped product is provided by the Taqman probe directed against the exon 47–52 junction, which only generates a fluorescent signal for the smaller product lacking exon 51(C). Gel analysis of qPCR products for Taqman assays that detect skipped transcripts (DMD ex44-52.3, DMD ex48-52, DMD ex49-52.2 & DMD ex50-53) and non-skipped transcripts (DMD ex51-53) in ex51 AON treated patient derived myotubes with different mutational backgrounds. DMD ex44-52.3, DMD ex48-52, DMD ex49-52.2 & DMD ex50-53 assays all show two products separated by ~233 bp (the size of exon 51): the transcript without exon 51 (lower band) and the transcript containing exon 51 (upper band). Specificity for the skipped product is provided by the Taqman probe which which only generates a fluorescent signal for the smaller product lacking exon 51. (TIF) [file pone.0162467.s001.tif]

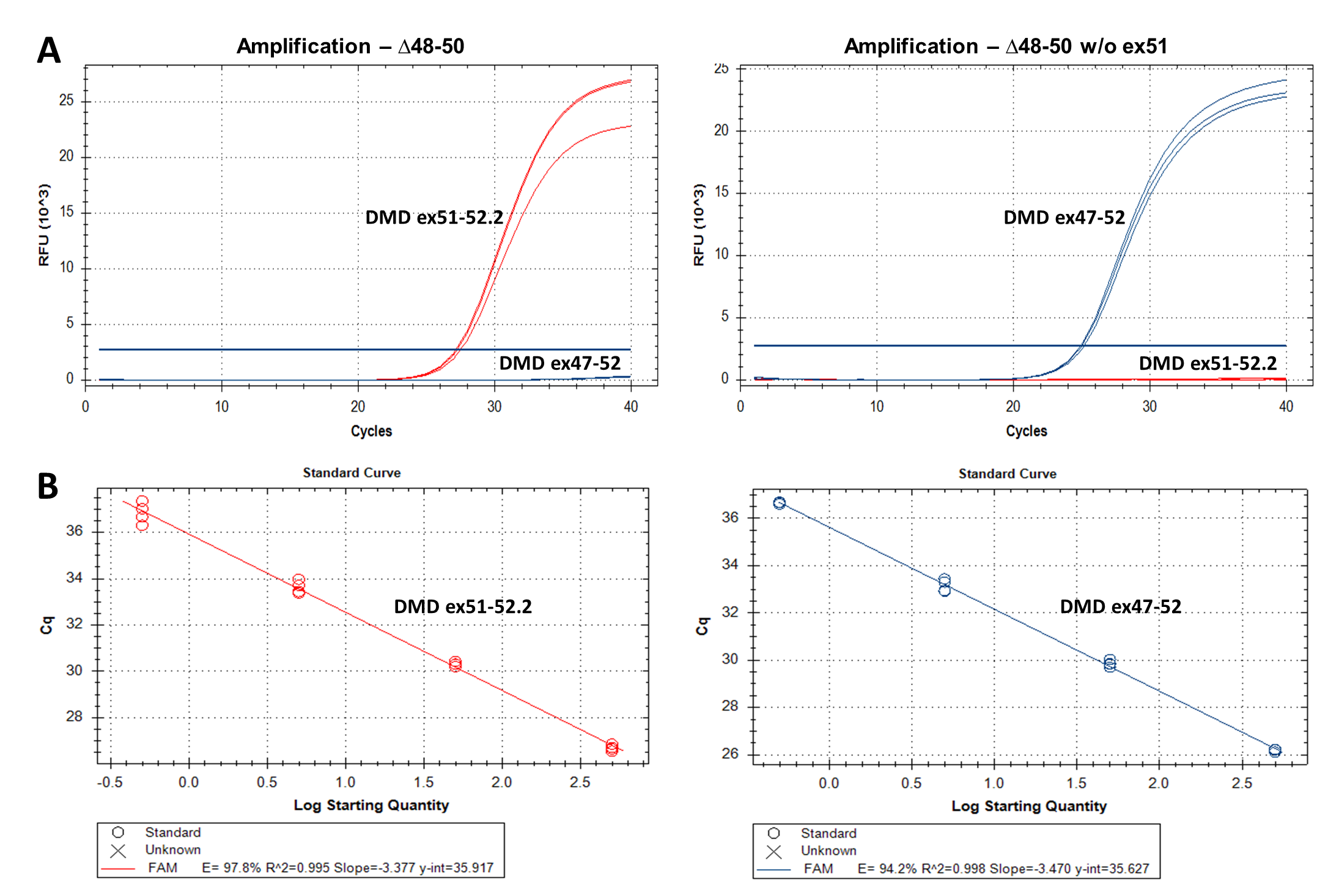

Supplement: S2 Fig — Specificity and efficiency of the Taqman assays representative for the exon 48–50 deletion transcripts in qPCR analysis. (A) The specificity of both DMD ex51-52.2 and DMD ex47-52 Taqman assays was confirmed by qPCR analysis using dystrophin cDNA constructs representative for Δ48–50 transcript fragments with or without exon 51. (B) 10-fold serial dilution of a 50–50 mixture of the dystrophin cDNA construct templates representative for Δ48–50 transcript fragments with or without exon 51. (TIF) [file pone.0162467.s002.tif]

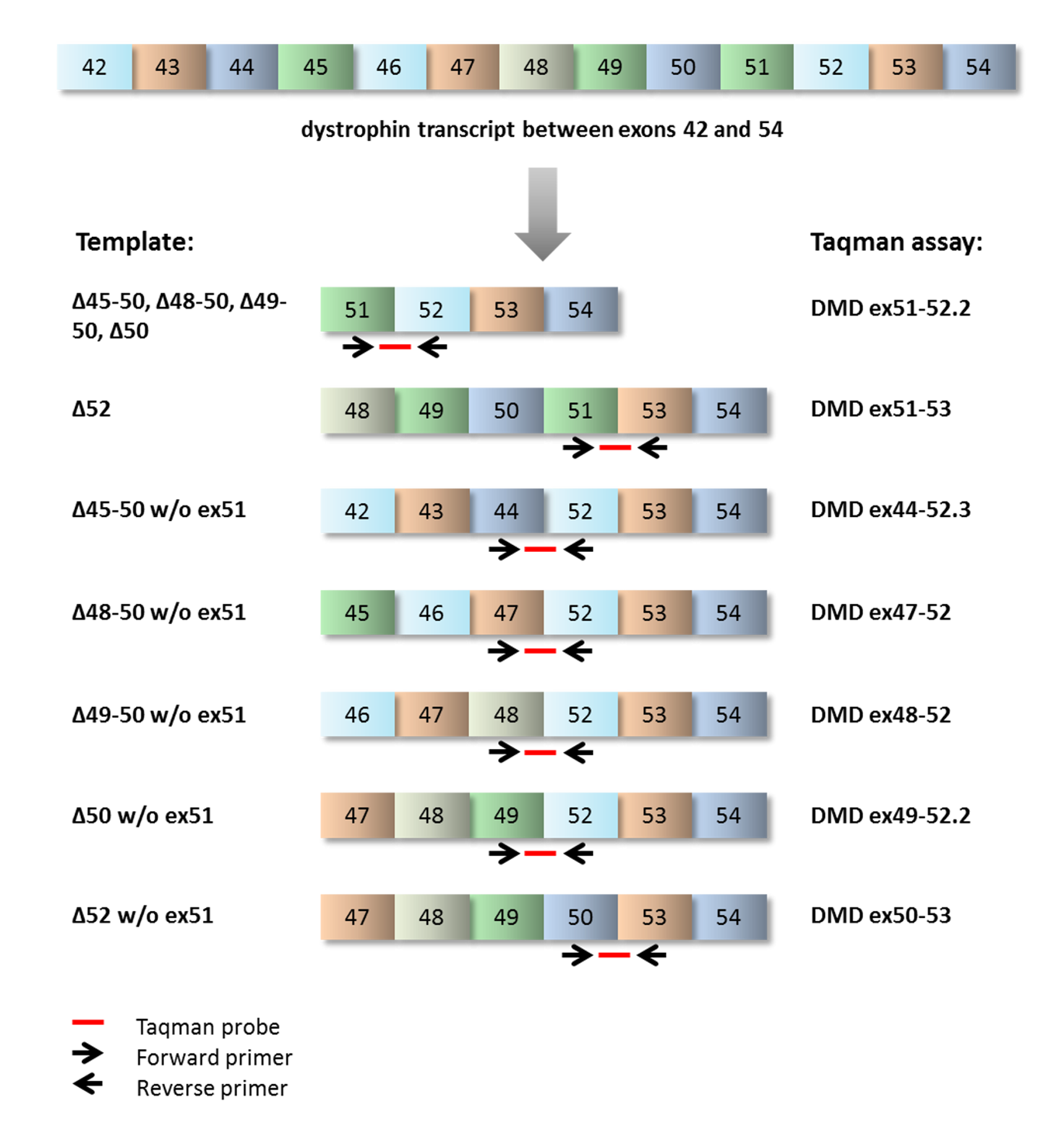

Supplement: S3 Fig — Taqman assays were designed to detect exon 51 skipping in samples of DMD patients with different relevant deletions using the guidelines of the Primer express software (Applied Biosystems). For the specific detection of transcripts containing exon 51, the forward primer was located in exon 51 and the reverse primer in exon 52, while the Taqman probe spans the exon-junction. For the samples with an exon 52 deletion, an alternative assay was designed to detect the transcript containing exon 51. (TIF) [file pone.0162467.s003.tif]
