# Supplementary material for: Digital Droplet PCR for the Absolute Quantification of Exon Skipping Induced by Antisense Oligonucleotides in (Pre-)Clinical Development for Duchenne Muscular Dystrophy
Source: PLoS One. 2016 Sep 9;11(9):e0162467. doi: 10.1371/journal.pone.0162467 (PMC5017733; doi:10.1371/journal.pone.0162467)
Supplement: S1 Table — Dystrophin cDNA constructs, representative for the transcripts arising from several exon 51 skip amenable deletions and the resulting transcript fragment following exon 51 skipping, were synthesized by Life Technologies. All cDNA constructs had a length of 1200bp and were verified by sequencing. Dark shaded exons were included entirely and light shaded exons were partially included. (PDF) [file pone.0162467.s004.pdf]

S1 Table. Design of dystrophin cDNA constructs for exon 51 skip amenable deletions

Vector ID	Exons included in vector															
	41	42	43	44	45	46	47	48	49	50	51	52	53	54	55	56
Δ45-50		178/195			-	-	-	-	-	-				138/155		
Δ45-50 w/o ex51	99/183				-	-	-	-	-	-	-				100/190	
Δ48-50				8/148				-	-	-						
Δ48-50 w/o ex51				124/148				-	-	-	-				117/190	
Δ49-50						146/148			-	-						
Δ49-50 w/o ex51					116/176				-	-	-				115/190	
Δ50						44/148				-						
Δ50 w/o ex51p						138/148				-	-				139/190	
Δ52							13/150					-				
Δ52 w/o ex51											-	-				96/173
