# Supplementary material for: Digital Droplet PCR for the Absolute Quantification of Exon Skipping Induced by Antisense Oligonucleotides in (Pre-)Clinical Development for Duchenne Muscular Dystrophy
Source: PLoS One. 2016 Sep 9;11(9):e0162467. doi: 10.1371/journal.pone.0162467 (PMC5017733; doi:10.1371/journal.pone.0162467)
Supplement: S2 Table — (PDF) [file pone.0162467.s005.pdf]

S2 Table. Sequences of PCR primers and Taqman probes

Deletion(s)	Assay	Forward primer	Reverse primer	Probe (FAM)	Product length (bp)
Δ45-50	skipped	CCTGAGAATTGGGAACATGCTAA (exon 44)	GACGCCTCTGTTCCAAATCC (exon 52)	ATACAAATGGTATCTTAAGGCAAC	73
Δ48-50	skipped	TGAAAATAAGCTCAAGCAGACAAATC (exon 47)	GACGCCTCTGTTCCAAATCC (exon 52)	CAGTGGATAAAGGCAACA	71
Δ49-50	skipped	CAACCAAACCAAGAAGGACCAT (exon 48)	GACGCCTCTGTTCCAAATCC (exon 52)	TGACGTTCAGGCAACA	64
Δ50	skipped	GGGCAGCATTTGTACAAGGAAA (exon 49)	GACGCCTCTGTTCCAAATCC (exon 52)	AGCCAGTGAAGGCAA	76
Δ52	skipped	CCTGACCTAGCTCCTGGACTGA (exon 50)	TGTACTTCATCCCACTGATTCTGAA (exon 53)	CTATTGGAGCCTTTGAAAG	71
Δ45-50, Δ48-50, Δ49-50, Δ50	non-skipped	GTGATGGTGGGTGACCTTGAG (exon 51)	GACGCCTCTGTTCCAAATCC (exon 52)	CAAGCAGAAGGCAACAA	82
Δ52	non-skipped	GTGACCTTGAGGATATCAACGAGAT (exon 51)	TGTACTTCATCCCACTGATTCTGAA (exon 53)	ATCAAGCAGAAGTTGAAAG	75
Δ48-50	Primary PCR	AGAACAAAAGAATATCTTGTCAG (exon 46)	TTGCCTCCGGTTCTGAAGG (exon 52)	n/a	WT: 702<br>w/o ex51: 469
	Nested PCR	CCCATAAGCCCAGAAGAGC (exon 47)	TCTAGCCTCTTGATTGCTGG (exon 52)	n/a	WT: 401<br>w/o ex51: 168
